# Biodistribution of Multimodal Gold Nanoclusters Designed for Photoluminescence-SPECT/CT Imaging and Diagnostic

**DOI:** 10.3390/nano12193259

**Published:** 2022-09-20

**Authors:** Greta Jarockyte, Marius Stasys, Vilius Poderys, Kornelija Buivydaite, Marijus Pleckaitis, Danute Bulotiene, Marija Matulionyte, Vitalijus Karabanovas, Ricardas Rotomskis

**Affiliations:** 1 Biomedical Physics Laboratory, National Cancer Institute, LT-08406 Vilnius, Lithuania; 2 Life Sciences Center, Vilnius University, LT-10257 Vilnius, Lithuania; 3 Department of Chemistry and Bioengineering, Vilnius Gediminas Technical University, LT-10223 Vilnius, Lithuania; 4 Laser Research Center, Faculty of Physics, Vilnius University, LT-10223 Vilnius, Lithuania

**Keywords:** imaging, multimodality, gold nanoclusters, luminescence, SPECT/CT, radiolabelling, technetium 99m, biodistribution, accumulation in cells

## Abstract

Highly biocompatible nanostructures for multimodality imaging are critical for clinical diagnostics improvements in the future. Combining optical imaging with other techniques may lead to important advances in diagnostics. The purpose of such a system would be to combine the individual advantages of each imaging method to provide reliable and accurate information at the site of the disease bypassing the limitations of each. The aim of the presented study was to evaluate biodistribution of the biocompatible technetium-99m labelled bovine serum albumin–gold nanoclusters (^99m^Tc-BSA-Au NCs) as photoluminescence-SPECT/CT agent in experimental animals. It was verified spectroscopically that radiolabelling with ^99m^Tc does not influence the optical properties of BSA-Au NCs within the synthesized ^99m^Tc-BSA-Au NCs bioconjugates. Biodistribution imaging of the ^99m^Tc-BSA-Au NCs in Wistar rats was performed using a clinical SPECT/CT system. In vivo imaging of Wistar rats demonstrated intense cardiac blood pool activity, as well as rapid blood clearance and accumulation in the kidneys, liver, and urinary bladder. Confocal images of kidney, liver and spleen tissues revealed no visible uptake indicating that the circulation lifetime of ^99m^Tc-BSA-Au NCs in the bloodstream might be too short for accumulation in these tissues. The cellular uptake of ^99m^Tc-BSA-Au NCs in kidney cells was also delayed and substantial accumulation was observed only after 24-h incubation. Based on our experiments, it was concluded that ^99m^Tc-BSA-Au NCs could be used as a contrast agent and shows promise as potential diagnostic agents for bloodstream imaging of the excretory organs in vivo.

## 1. Introduction

Early and precise tumour detection is one of the most important keys to successful elimination of cancerous diseases. In many cases of malignant tumours, non-invasive determination of the disease spreading in the body can be a problematic task for either oncologists or surgeons. In recent years, advances in imaging instruments, imaging probes, and quantification techniques have enabled more refined and better-quality images for more accurate diagnoses. Molecular imaging can be defined as in vivo visualization, characterization and measurement of biological processes at the molecular and cellular levels [1,2]. Various molecular imaging modalities have been exploited for cancer diagnosis and treatment assessment. The commonly used molecular imaging techniques include computed tomography (CT), positron emission tomography (PET), single photon emission computed tomography (SPECT), molecular magnetic resonance imaging (MRI), contrast-enhanced ultrasound (CEU), optical bioluminescence, and optical fluorescence [3,4,5,6]. Each imaging method has its own advantages and limitations. Radionuclide-based imaging techniques, such as PET and SPECT, are highly sensitive and quantitative, but they have relatively poor spatial resolution. MRI provides high spatial resolution images with exquisite soft tissue contrast, yet it suffers from low sensitivity. Meanwhile, optical imaging can sensitively and sequentially interrogate cellular and molecular functions in living subjects; however, the energies in the visible (VIS) to near-infrared region (NIR) of the spectrum are limited to penetrate the depth of mammalian tissues. Therefore, combinations of imaging techniques, as so-called “multimodality imaging”, are being designed to take advantage of the strengths of modalities while minimizing their limitations, which, as a result, may simultaneously provide comprehensive biological information [7,8]. The prospect of combining optical with other imaging methods has drawn attention, and it could lead to important advances in clinical diagnostics [6]. Multimodality imaging is aimed at detecting progress of various diseases, biodistribution of drugs, assessing metabolic changes, and molecular events in vivo [9]. Such approach would provide additional pathological information to the physicians compared with single modality methods and might help to shorten the patient’s pathway to recovery. For each of the currently existing diagnostic imaging methods (optical, MRI, X-ray), a development of more sensitive and specific contrast agents is a major scientific and clinical goal.

Progress of nanomaterial science provides the ability to design multifunctional imaging agents that could be used for several imaging techniques. Among the wide spectrum of nanomaterials currently in use, gold nanoclusters (Au NCs) have drawn a lot of attention in scientific community as promising agents for cancer diagnostics and therapy [10,11,12,13,14,15,16]. Au NCs have absorption and photoluminescence (PL) in the VIS spectral region [10,12], as well as strong X-ray absorption [11,17], which makes them good candidates for dual-modality imaging [18]. Fang et al. demonstrated that specifically targeted Au NCs might be used not only for bioimaging but also to enhance the radiosensitization effect, resulting in cancer cells’ death in vitro [19]. Moreover, Au NCs have been shown as effective photosensitizers for photodynamic tumour therapy [20,21].

Protein stabilized Au NCs are especially promising due to their eco-friendly green synthesis and biocompatibility [10,20,22,23]. Since Xie et al. demonstrated Au NCs stabilization with bovine serum albumin (BSA) [10], various proteins were used for Au NCs synthesis [24], although BSA-Au NCs still remain one of the most common choices. BSA-Au NCs are investigated as potential photoluminescent labels for cancer detection [25,26,27,28]. However, due to limitation of excitation light penetration into deeper tissues and low PL quantum yield, BSA-Au NCs are more suitable for superficial imaging as optical tracking of such NCs in vivo is complex and requires extra efforts. BSA-Au NCs could be used as a multimodality imaging agents, without any additional modifications. For example, it was demonstrated that BSA-Au NCs could be used for diagnostic of renal abnormalities with computerized tomography (CT): 2 h post intravenous injection, BSA-Au NCs were detected in the renal collecting system of mice [29]. In addition, the zwitterion functionalized Au NCs show potential to efficiently absorb NIR light and generate photoacoustic signals in vivo [30]. However, in some cases extra modifications are necessary due to a peculiarity of a specific method. BSA-Au NCs functionalization with gadolinium (Gd) allows to use them for MRI [28,31,32]. The iodine-124 labelled gold nanoclusters showed potential as imaging agents for positron emission tomography of lung tumours [33].

SPECT is a common diagnostic method used for precise tumour diagnostics, three-dimensional (3D) imaging of organs or organ-systems as well as blood flow and metabolism examination in the body. The technique requires the injection of a gamma-emitting radionuclide into the patient’s bloodstream. Currently, the most-used radionuclides for SPECT are ^99m^Tc (t_1/2_ = 6 h) and ^111^In (t_1/2_ = 2.8 days) [34]. SPECT is frequently combined with CT, which lends itself to a wide range of useful diagnostic applications and has a well-established clinical impact in most cases [34,35]. It was demonstrated, that ^99m^Tc SPECT/CT improves the detection rate of cervical [36], endometrial [37] and other locations’ [35] cancers compared to lymphoscintigraphy. Derivatives of the serum albumin, radiolabelled with ^99m^Tc have already been applied in routine clinical practice [38].

Due to their characteristic optical and gamma radiation emitting properties, we have chosen BSA-Au NCs and ^99m^Tc for creating the biocompatible multifunctional imaging tracer for SPECT/PL dual imaging. The purpose of this study was to evaluate accumulation and distribution of ^99m^Tc-BSA-Au NCs in experimental animals and assess their suitability as dual-imaging probes for in vivo imaging. 

## 2. Materials and Methods

### 2.1. ^99m^Tc-BSA-Au NCs Synthesis

**Figure 1:**
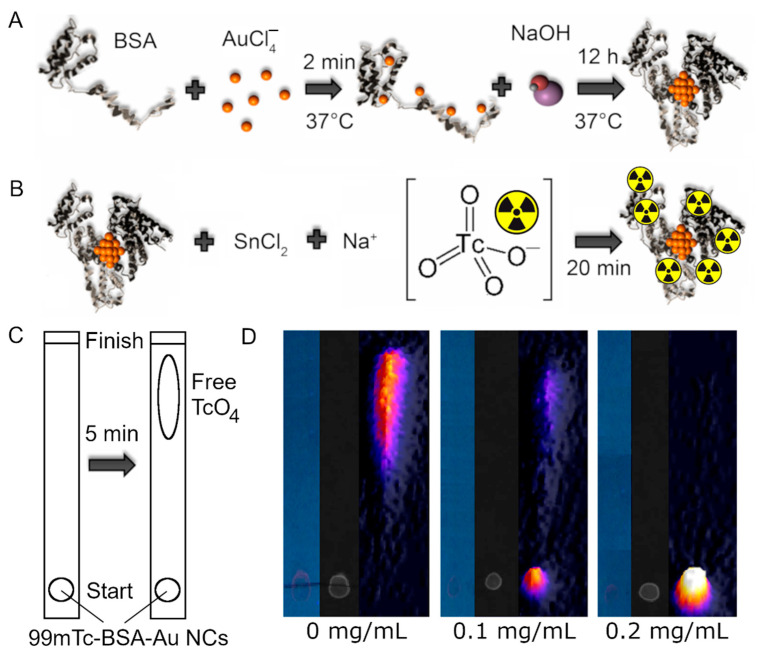
(**A**)—BSA-Au NCs synthesis: tetrachlorauric acid (HAuCl_4_) was mixed with BSA and then sodium hydroxide (NaOH) was added as a reducing agent in the final step. (**B**)—Radiolabelling was performed by mixing BSA-Au NCs with the stannous chloride (SnCl_2_) aqueous solution and sodium pertechnetate (NaTcO_4_). (**C**)—Graphical illustration of ^99m^Tc-BSA-Au NCs thin-layer chromatography. Samples prepared with different SnCl_2_ concentrations were applied onto chromatography paper strips and then placed in 99% acetone solution for 5 min. (**D**)—Chromatography results of ^99m^Tc binding to BSA-Au NCs at various SnCl_2_ concentrations. From left to right: VIS picture under ultraviolet (UV) light, NIR PL image, nuclear medicine static images aligned with the thin-layer chromatography strips used for radiochemical quality control of the ^99m^Tc-BSA-Au NCs solutions with varied concentration of SnCl_2_ used for the labelling: 0 mg/mL, 0.1 mg/mL, 0.2 mg/mL.

BSA-Au NCs were synthesized according to [10] by mixing tetrachlorauric acid (HAuCl_4_) (Sigma-Aldrich, St. Loui, MO, USA) with BSA (Sigma-Aldrich, St. Loui, MO, USA) and then adding sodium hydroxide (NaOH) (Sigma-Aldrich, St. Loui, MO, USA) as a reducing agent in the final step (Figure 1A). 

To prepare dual-imaging probe, BSA-Au NCs were radiolabelled with 99mTc. Briefly, 1 mL 24 mg/mL BSA-Au NCs aqueous solution was mixed with the stannous chloride (SnCl_2_) (Sigma-Aldrich, St. Loui, MO, USA) solution (4 mg of SnCl_2_ salt (Sigma-Aldrich, St. Loui, MO, USA) was dissolved in 10 mL of saline (NaCl) (0.9%)) according to [38,39] (Figure 1B). The optimal amount of SnCl_2_ was determined using the thin-layer chromatography (TLC) (Whatmann 3 MM chromatography paper (Sigma-Aldrich, St. Loui, MO, USA) was used as a stationary phase and 99% acetone (Sigma-Aldrich) as a mobile phase). 2.0 GBq of sodium pertechnetate (NaTcO_4_) was added to the SnCl_2_ and BSA-Au NCs mixture (Figure 1B). 99mTc was eluted from a Molybdenum-99/Technecium-99m generator using 0.9% NaCl solution (GE Healthcare, Chicago, IL, USA). The radioactivity was measured by dose calibrator (VDC-404, Veenstra Instruments, Ahlerstedt, Germany). Chromatographic strips were scanned using a Siemens Symbia T6 gamma camera with a Low Energy High Resolution (LEHR) collimator, placing a strip on the detector, and acquiring a 5 min static image. Quantitative analysis of the images based on the counts in regions of interest (ROIs) was carried out using ImageJ software [40].

### 2.2. Radiochemical Yield

The quality of radiolabelling was evaluated using TLC. Schematic illustration of procedure is presented in Figure 1C. The radiochemical purity was calculated by measuring the counts corresponding to the free pertechnetate and the overall activity from the strip [31]. As it can be seen from the chromatograms presented in Figure 1D, the sufficient amount of tin chloride for radiolabelling of BSA-Au NCs was 0.2 mg/mL. 

The calculated radiochemical purity resulted in above 95%. Visualization of the BSA-Au NCs photoluminescence in ^99m^Tc-BSA-Au NCs complex on the TLC strips was performed employing an IBOX^®^ UV lamp with an excitation band-pass filter (peak at 400 nm) and registered using a CCD camera with a pass-filter in the spectral region 615–685 nm. 

The stability of the radiolabelled nanoparticles was checked by repeating the TLC test over a 2 h period. Testing at 2 h post-labelling, yielded a radiochemical purity of over 95% (comparable to that obtained initially), indicating that imaging can be performed through at least 2 h (see Supplementary Materials Figure S1).

### 2.3. Optical Spectroscopy Measurements

Absorption spectra were measured using a Cary50 (Varian. Inc., Australia) spectrometer (slit bandwidth 1.5 nm, step—1 nm). Photoluminescence (excitation wavelength 405 nm) and PL excitation (emission wavelength 650 nm) spectra of BSA-Au NCs and ^99m^Tc-BSA-Au NCs were measured using a Cary Eclipse (Varian Inc.) fluorescence spectrometer (excitation and emission slits 5 nm, step—1 nm). Spectroscopic measurements in the visible spectral region were performed using aqueous BSA-Au NCs and ^99m^Tc-BSA-Au NCs solutions of 24 mg/mL and 6 mg/mL concentrations, respectively. Due to the high optical density of proteins in UV region, absorbance measurements were performed using 10-times lower concentrations. Quartz cells (Hellma Optik GmbH, Germany) with an optical path length of 1 cm were used for all optical measurements.

### 2.4. Animal Model

All animal procedures were performed in accordance with the guidelines established by State Food and Veterinary Service Animal Care and Use Committee (Vilnius, Lithuania) that approved the current study (approval No. G2-156, approved 15 September 2020). Albino Wistar rats (9 weeks old, approx. 185 ± 11 g, female, n = 9) were obtained from the State Scientific Research Institute of Innovative Medical Center (Vilnius, Lithuania). Animals were housed under conditions of constant temperature, humidity, and standard light/dark cycle. Food and fresh drinking water were available *ad libitum*. Animals were acclimated for at least 7 days before the experiments.

### 2.5. In Vivo Imaging

All animals were anesthetized and positioned supine for the experiments in vivo. Experimental animals (n = 6) received an injection of 150 MBq in 0.2 mL of ^99m^Tc-BSA-Au NCs (6.5 mg/kg) into a tail vein. Additionally, for control animals (n = 3) BSA was dissolved in 0.9% saline and 0.2 mL of BSA-saline solution (6.5 mg/kg). 

Siemens Symbia T6 clinical SPECT/CT dual-headed gamma camera with Low Energy High Resolution (LEHR) collimators was used for planar, dynamic and SPECT/CT studies. The dynamic acquisition was started 1 min after the injection of the selected radiopharmaceutical. The gamma camera energy window was set to 140 keV +/−10%. Dynamic imaging parameters were as follows: 256 × 256 matrix; zoom 3.2, 360 frames, single frame duration 15 s. Static image settings: 256 × 256 matrix; zoom: 3.2, single frame duration 20 s. SPECT acquisition parameters: 256 × 256 matrix; zoom 3.2, 15 s per projection; and 90 projections.

All CT images were recorded using the same Siemens Symbia T6 Hybrid system. CT acquisition parameters: slice thickness 1.25 mm, X-ray tube voltage 120 kVp, X-ray tube rotation speed 0.8 s per rotation, and tube current 100 mA. CT FOV corresponding to the full SPECT FOV.

CT and SPECT images were merged using the Siemens MI Syngo multi-image viewing software. SPECT/CT images were used for anatomical localization to identify the regions of functional accumulation of the tracer compared with planar studies.

### 2.6. Nuclear Medicine Imaging Quantification

Dynamic nuclear medicine images were analysed using Siemens MI Apps software dedicated to renography studies. Regions of interest were drawn around certain regions on an image corresponding to a specific organ or a group of organs according to a rat’s atlas and CT images. Time–activity (kCounts) curves were generated for all ROIs, considering decay correction, and are presented as gamma intensity rate in kCounts per second vs. min. 

### 2.7. Histology

Immediately after SPECT/CT imaging (2 h post injection, n = 3) or the next day (24 h post injection, n = 3), experimental animals were euthanized, and organs dissected. Histological sections of the organs were prepared by freezing the organs and using standard staining procedure with haematoxylin-eosin (H&E). Mirrored sections of each sample were left unstained and used for a confocal laser scanning microscopy (Nikon EclipseTE2000 C1si confocal microscope, Nikon, Tokyo, Japan) to determine ^99m^Tc-BSA-Au NCs localization in the kidneys, liver, and spleen. Photoluminescence signal in tissues was evaluated with 488 nm argon laser excitation. 

### 2.8. Laser Scanning Confocal Microscopy

The accumulation and distribution of ^99m^Tc-BSA-Au NCs in histological section of the organs and kidney cells were assessed using the Nikon EclipseTE2000 C1si confocal microscope (Nikon, Japan) equipped with a diode laser for 404 nm wavelength excitation and an argon ion laser for 488 nm excitation. Imaging was performed using 10×/0.25 NA and 20×/0.50 NA objectives (Nikon, Japan) and 60×/1.4 NA oil immersion objective (Nikon, Japan). The three-channel RGB detector filters (band-pass filters 450/17, 545/45 and 688/67 for blue, green, and red channels, respectively) were used. Image processing was performed using the Nikon EZ-C1 Bronze version 3.80 and ImageJ 1.8.0_172 software.

### 2.9. Cell Culturing

Embryonic human kidney cells HEK-293T were used for cellular experiments. Cells were cultured in a cell growth medium (DMEM, Corning, New York, NY, USA), supplemented with 10% (*v/v*) foetal bovine serum (Gibco, New York, NY, USA), 100 U/mL penicillin and 100 µg/mL streptomycin (Gibco, USA) and 1% MEM Non-Essential Amino Acids (Gibco, USA). Cells were maintained at 37 °C in a humidified atmosphere containing 5% of CO_2_. The cells were routinely subcultured 2–3 times a week in 25 cm^2^ cells’ culture flasks (TPP, Switzerland).

### 2.10. Cytotoxicity 

HEK-293T cells were seeded on a 96-wellplate (TPP, Switzerland) at a density of 1.2 × 10^3^ cells/well. After 24 h, the old medium was replaced with a fresh medium containing 1.5, 3, 7.5 or 15 mg/mL ^99m^Tc-BSA-Au NCs, while medium alone without ^99m^Tc-BSA-Au NCs was a control. Cells were incubated for 24 h in the dark. The next day, Pierce LDH Cytotoxicity Assay Kit (Pierce Biotechnology, Thermo Scientific, Eugene, OR, USA) was used to detect extracellular appearance of lactate dehydrogenase (LDH). The concentration of extracellular LDH was quantified by measuring absorbance at 490 and 630 nm wavelength with plate-reading spectrophotometer (BioTek, Winooski, VT, USA). After obtaining absorbance values, they were recalculated according to the protocol to represent percentage values of cytotoxicity. For better data representation, estimated cytotoxicity (%) was recalculated to viability of cells (100%—cytotoxicity (%)). Data are expressed as mean ± standard deviation (SD). The statistical significance of differences between studied groups was assessed using a two-tailed independent Student’s *t*-test at the 95% confidence level. The level of statistical significance was expressed as *p*-values < 0.05.

### 2.11. Accumulation in Cells

For intracellular imaging studies, HEK-293T cells were seeded into an 8-chambered cover glass plate (ThermoFisher, Rochaster, NY, USA) with a density of 3 × 10^4^ cells/chamber. For the evaluation of ^99m^Tc-BSA-Au NCs uptake and intracellular localization, cells were treated with 15 mg/mL of ^99m^Tc-BSA-Au NCs and incubated for different periods of time. Nuclei of the cells were stained with 0.01 mg/mL Hoechst 33258 (Sigma-Aldrich, Germany). The accumulation of ^99m^Tc-BSA-Au NCs was observed using confocal laser scanning microscopy, as describe before. Hoechst 33258 was excited at 404 nm, ^99m^Tc-BSA-Au NCs was excited at 488 nm. During imaging the cells were incubated at 37 °C in the Microscope Stage Incubation System (OkoLab, Pozzuoli, Italy) in a humidified atmosphere containing 5% of CO_2_ (0.80 Nl/min O_2_ and 0.04 Nl/min CO_2_). 

Additionally, accumulation dynamics of ^99m^Tc-BSA-Au NCs in HEK-293T cells was spectroscopically evaluated using Edinburgh spectrometer FLS920, measuring emission intensity of cell suspensions. Accumulation dynamics is determined as integral of emission intensity (600–700 nm) at the specific time divided by cell number at that time in the suspension and plotted as emission intensity per cell. Experiments with cells were performed in triplicate and repeated three times. Data are expressed as mean ± standard deviation (SD).

## 3. Results and Discussion

### 3.1. Characterisation

**Figure 2:**
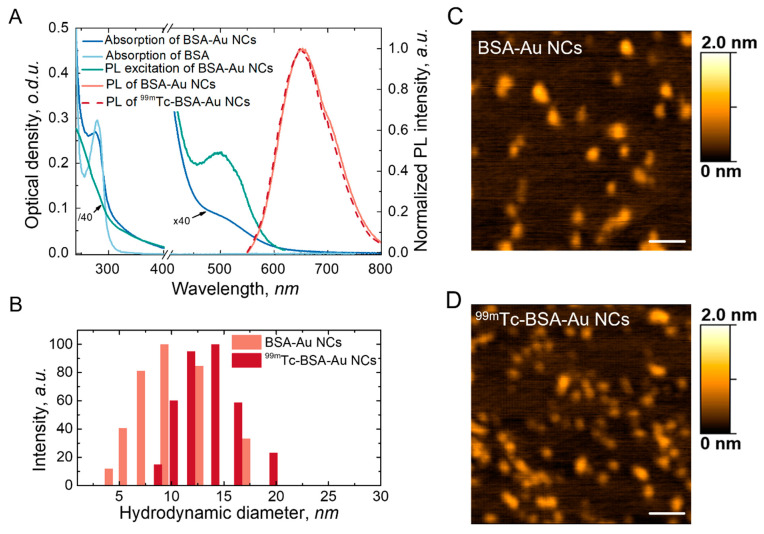
Spectroscopic analysis of BSA-Au NCs and ^99m^Tc-BSA-Au NCs (**A**), histogram of hydrodynamic diameter of BSA-Au NCs and ^99m^Tc-BSA-Au NCs (**B**), AFM images of BSA-Au NCs (**C**) and ^99m^Tc-BSA-Au NCs (**D**), scale bars correspond to 100 nm.

Optical and spatial characteristics of BSA-Au NCs were measured and displayed in Figure 2. Absorption, PL and PL excitation spectra of freshly synthesized BSA-Au NCs are presented in Figure 2A. Absorption of BSA-Au NCs increases in the short wavelength region and has an absorption band around 280 nm (Figure 2A, blue line), which coincides with to the absorption band of pure BSA (Figure 2A, light blue line), indicating that 280 nm wavelength is mainly absorbed by the protein part of BSA-Au NCs. The PL band of the BSA-Au NCs (ex. at 405 nm) have a maximum around 650 nm (Figure 2A, light red line). BSA-Au NCs PL excitation spectrum have a gradual slope from UV to the longer wavelength region with a slope at 510 nm (Figure 2A, green line). PL in the red spectral region makes the BSA-Au NCs a promising PL marker for biological tissues because of the biological tissue transparency window in the red and NIR spectral region. 

BSA-Au NCs radiolabelling with 99mTc did not affect the optical properties of the BSA-Au NCs. ^99m^Tc-BSA-Au NCs have characteristic PL band with a peak around 650 nm (Figure 2, red dashed line) which coincide with the PL band of the BSA-Au NCs (Figure 2A, red line) The fact that the shape of the PL curve is not influenced by labelling retains the possibility of optical detection in a clinical environment. Due to characteristic optical and gamma radiation emitting properties, multifunctional imaging tracer ^99m^Tc-BSA-Au NCs could be used for SPECT/PL dual imaging.

AFM images of BSA-Au NCs and ^99m^Tc-BSA-Au NCs did not reveal significant differences between BSA-Au NCs and ^99m^Tc-BSA-Au NCs. It is reported that during synthesis of the BSA-Au NCs a small cluster consisting of 25 gold atoms is formed inside the BSA molecule [10,21]. It was demonstrated that formation of gold nanoclusters within BSA leads only to a slight increase in size: the hydrodynamic diameter of the BSA-Au NCs is 8.4 nm compared to 6.8 nm in case of the BSA [12,21]. Dynamic light scattering results showed that attaching of the ^99m^Tc to the BSA-Au NCs increases the hydrodynamic diameter from 9 nm to 13 nm of ^99m^Tc-BSA-Au NCs. The increase in NCs could appear due to BSA conformation changes after ^99m^Tc attachment [41,42].

In our previous study we demonstrated that BSA-Au NCs are colloidally stable for more than a month in stock solution after synthesis [12]. Additionally, BSA-Au NCs remained stable for at least for 24 h in 10% FBS buffer solution [29]. However, there is not enough information about ^99m^Tc-BSA-Au NCs stability in biological media; stability of ^99m^Tc-BSA-Au NCs in Wistar rat’s blood plasma was investigated before moving on with in vivo experiments. Our result showed that ^99m^Tc-BSA-Au NCs are stable in rat’s blood plasma, at least for 3 days (see Supplementary Materials Figure S2), and are suitable for in vivo studies. Additionally, for ^99m^Tc-BSA-Au NCs, dispersed in plasma, binding stability was evaluated and compared to stock solutions. There were no differences observed comparing binding stability of stock solution and ^99m^Tc-Au-BSA NCs diluted in Wistar rat’s blood plasma (see Supplementary Materials Figure S1). 

### 3.2. Dynamic Analysis of the ^99m^Tc-BSA-Au NCs Biodistribution In Vivo

**Figure 3:**
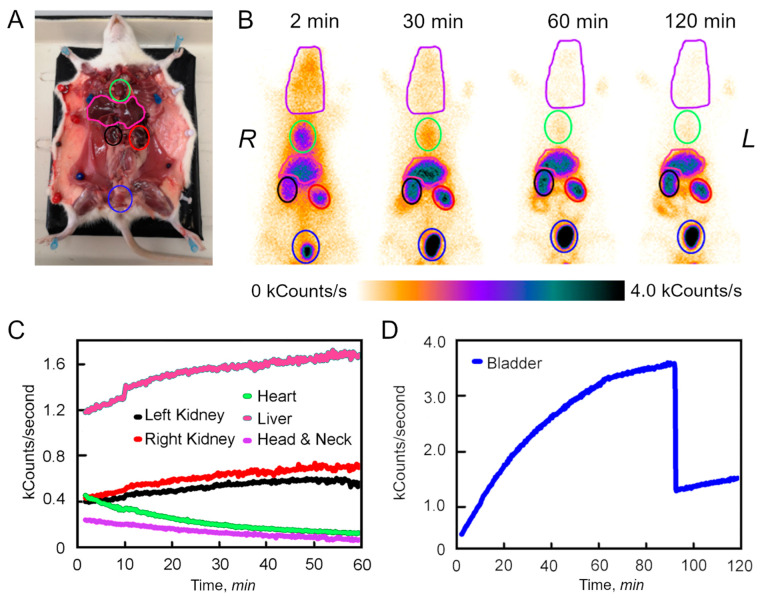
(**A**)—Anatomy of Wistar rat. Main organs marked as ROIs: heart (green), liver (pink), left kidney (black), right kidney (red), bladder (blue). (**B**)—Anterior planar nuclear medicine images acquired respectively at 2, 30, 60 and 120 min p.i. On the static planar images, the black colour represents the highest intensity, and orange, correspondingly, the lowest intensity. The head and neck regions were included for the purposes of quality control. (**C**)—Activity vs. time curves of the selected ROIs on planar dynamic images. (**D**)—Activity vs. time curves of the bladder ROI on planar dynamic images.

Distribution dynamic of ^99m^Tc-BSA-Au NCs in experimental animals (at 2, 30, 60 and 120 min post injection (p.i.)) are presented in Figure 3. Within the first few minutes the radioactivity signal from ^99m^Tc-BSA-Au NCs was detected all over the body of the experimental animals. It seems that radioactivity signal detected corresponds to the distribution of ^99m^Tc-BSA-Au NCs in the blood stream. The highest signal intensity of ^99m^Tc-BSA-Au NCs was detected in the organs, which have higher volume of the blood (heart, lung, liver, kidneys) and head, neck and bladder regions. Rapid decrease in the radioactivity signal of the ^99m^Tc-BSA-Au NCs 30 min p.i. was detected all over the bodies of rats. Reduced intensity in lung, head and neck regions was observed indicating the rapid clearance of the ^99m^Tc-BSA-Au NCs from the blood vessels and moving to the liver, kidney and the bladder. Within 60 min of the monitoring, the heart region exhibited over time decreasing radioactivity intensity, whereas in the liver region, as well in the kidneys and the bladder, the radioactivity signal intensity continued to increase. At 60 min p.i., notable radioactivity signal intensity appeared in the intestine region. At 120 min p.i., the detectable radioactivity signal remained in the bladder, liver and both kidneys, low signal was registered in the intestine region, and no radioactivity signal was observed in the chest, head and neck regions. The highest radioactivity signal intensity was detected in the bladder region. Such findings indicate the urinary tract as one of the possible pathways for excretion of the ^99m^Tc-BSA-Au NCs.

Following the accumulation experiments, the regions of interest assigned for particular organs (head and neck ROI, heart ROI, liver ROI, left kidney ROI, right kidney ROI, bladder ROI) were identified (Figure 3B) and were used for dynamic analysis (Figure 3C-D). The radioactivity signal of the ^99m^Tc-BSA-Au NCs from the head and neck region gradually decreased for at least 2 h p.i. A similar tendency was observed in the heart region. As the concentration of the tracer in the heart, head and neck regions was decreasing, the kinetic measurements showed an increase in the radioactive signal intensity in ROIs of kidneys and liver until it reached plateau at 60 min. However, as it can be seen from Figure 3D, the radioactivity signal intensity in the bladder continued to increase further. After emptying the bladder at ~90 min p.i., the radioactivity signal intensity in the bladder ROI started increasing again until the end of the measurement (Figure 3D).

The relatively high uptake of ^99m^Tc-BSA-Au NCs in the urinary system suggests excretion of ^99m^Tc-BSA-Au NCs to the bladder. The hydrodynamic diameter of ^99m^Tc-BSA-Au NCs is 13 nm (Figure 2). The glomerular slit pore size was originally reported to be a rectangular pore approximately 4 by 14 nm in cross section and 7 nm in length [43]; and by an electron tomography study it has been revealed that the glomerular slit-pores are 3.5 nm in diameter with some variation in size [44]. Nevertheless, it has been shown that some fraction of albumin could pass through the slit pores in normal rats, probably because of their flexibility and ellipsoid shape [45]. In the other literature, albumin molecules are reported to be taken up into lysosomes in the proximal tubule within 6 to 15 min and then degraded to amino acids during renal passage probably by tubular cells after 30 to 120 min in the proximal tubule [46,47]. Therefore, as ^99m^Tc-BSA-Au NCs are comparable in size to the glomerular slit pore, thus ^99m^Tc-BSA-Au NCs could have been excreted through the slit pores. That could explain the detected activity in the bladder. It is known that to some extent albumin is degraded to amino acids in the kidneys, yet it does not contradict the hypothesis that the radiolabelled portion of ^99m^Tc-BSA-Au NCs can get excreted to the bladder as is observed during our experiments. The results of the distribution in vivo would suggest that ^99m^Tc-Au-BSA kinetic pathways correspond with the results presented in [48]. Zhang et al. showed that BSA-Au NCs aggregates (40–80 nm) accumulates mainly in the liver and spleen, whereas smaller GSH-Au NCs (5–30 nm) are cleared through the urinary tract. Additionally, as it can be seen from Figure 3A, up to 1 h p.i. minor accumulation of ^99m^Tc-BSA-Au NCs was detected in the gut. This could be due to the alternative mechanism of the excretion pathway of the tracer, allowing for ^99m^Tc-BSA-Au NCs to be potentially used for protein-losing gastroenteropathy as has been established for ^99m^Tc-HSA [49,50]. Moreover, ^99m^Tc-BSA-Au NCs could also be employed as a tracer for lymphoscintigraphy as the conventional ^99m^Tc-HSA [45]. It would add value to the morphological in vitro investigation of the node as a cross-check confirmation of the in vivo findings. As a multimodal tracer, ^99m^Tc-BSA-Au NCs could add useful optical information to the diagnostics, in comparison with the conventional serum albumin-based radiopharmaceuticals [51,52]. 

### 3.3. ^99m^Tc-Au-BSA NCs Localization

**Figure 4:**
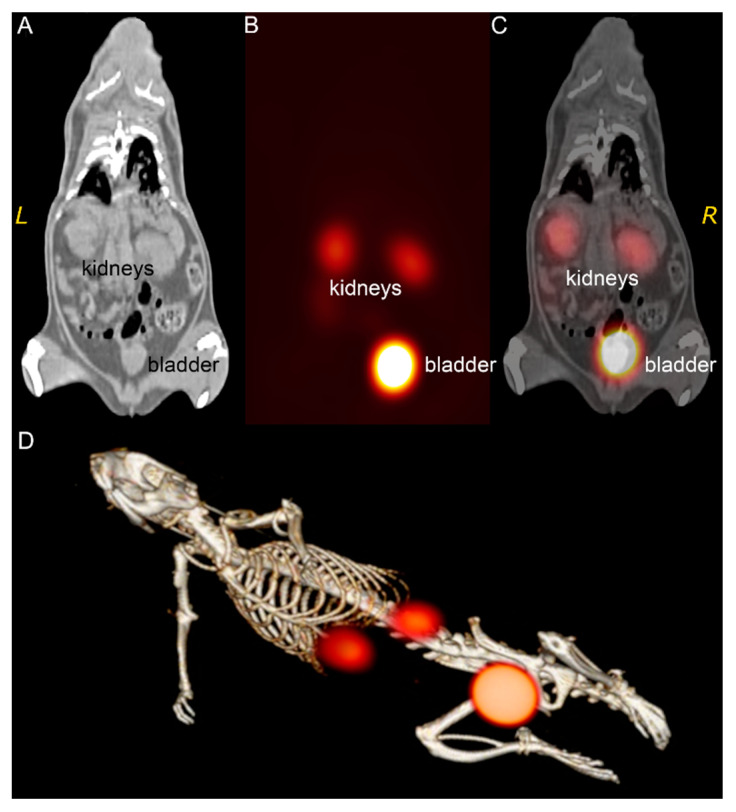
CT, SPECT and fused SPECT/CT coronal image of ^99m^Tc-BSA-Au NCs distribution in the animal model at 90 min p.i. (**A**)—CT image with clear anatomical visualization of kidneys. (**B**)—SPECT coronal slice with the visible kidneys and bladder regions. (**C**)—Fused images giving a more precise localization of the tracer uptake in the organ areas seen on planar images. (**D**)—3D volume rendered image using the data from (**A**–**C**).

In order to precisely evaluate the localization of ^99m^Tc-BSA-Au NCs after intravenous injection, SPECT/CT imaging was performed. SPECT imaging was utilized to examine in vivo trafficking of ^99m^Tc-BSA-Au NCs and CT scan was used to visualize anatomical structures of Wistar rat. In vivo SPECT/CT imaging (Figure 4) demonstrated that after 90 min p.i. the highest uptake of ^99m^Tc-BSA-Au NCs was in the kidneys and the bladder. All other organs showed a minimal uptake. It seems that distribution of ^99m^Tc-BSA-Au NCs throughout the experimental animal body is similar to the behaviour of ^99m^Tc-HSA [38,39], contrary to the accumulation of the free pertechnetate which is known to be accumulated mainly in thyroid and salivary glands [53]. In addition, from our results it could be concluded that gold nanoclusters formed inside the albumin do not affect the behaviour of the albumin itself in vivo. specific accumulation in kidney and bladder could be useful for the early diagnostics and treatment of these organs’ tumours; however, the possible toxicity of ^99m^Tc-BSA-Au NCs must be considered before these nanoclusters are used in a clinical environment.

### 3.4. Histology

**Figure 5:**
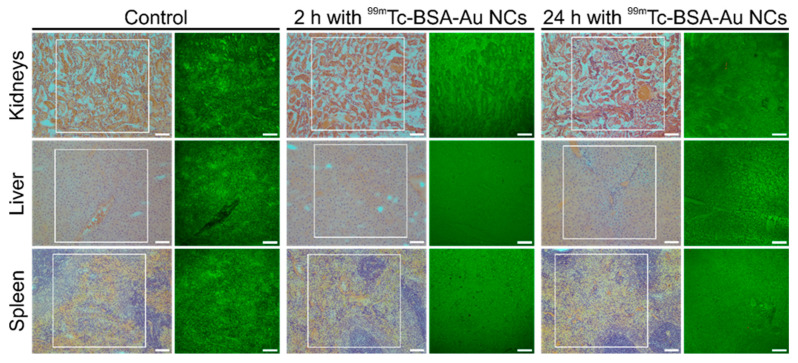
Representative H&E staining and confocal microscopy ex vivo images showing distribution of ^99m^Tc-BSA-Au NCs in the kidney, liver and spleen (2h and 24 h p.i.) of Wistar rat, which was intravenously injected with ^99m^Tc-BSA-Au NCs (6.5 mg/kg body mass) into the tail vein. Control rats received 6.5 mg/kg BSA dissolved in saline injection. Green colour—autofluorescence signal, red—photoluminescence of ^99m^Tc-BSA-Au NCs. Scale bars for all images—100 µm.

During ex vivo histology assessment of the rat organs by SPECT imaging, ^99m^Tc-BSA-Au NCs were detected in kidneys, liver and spleen. Two target times were chosen: 2 h and 24 h p.i. into the tail vein. Figure 5 shows standard H&E-stained images of organs from control and ^99m^Tc-BSA-Au NCs injected rats. H&E images were taken with a conventional bright field microscope at a magnification of 10×. The unstained, mirrored slices of tissues were imaged with a laser scanning confocal microscope at a magnification of 20×. In green channel (500–590 nm) auto-fluorescence signal from sections of organs was detected, while in red channel (620–755 nm) PL signal from gold nanoclusters was depicted. However, as it is seen in Figure 5, no signal of ^99m^Tc-BSA-Au PL in red channel was detected, or it was lower than auto-fluorescence in red channel. Our results demonstrate that ^99m^Tc-BSA-Au NCs penetration from blood vessels to tissues are probably limited or very slow. Since ^99m^Tc-BSA-Au NCs clearance from blood is fast—^99m^Tc-BSA-Au NCs quickly are filtered through kidneys to urine—presumably ^99m^Tc-BSA-Au NCs circulation in bloodstream time is too short for accumulation in tissues. 

### 3.5. Cellular Studies

**Figure 6:**
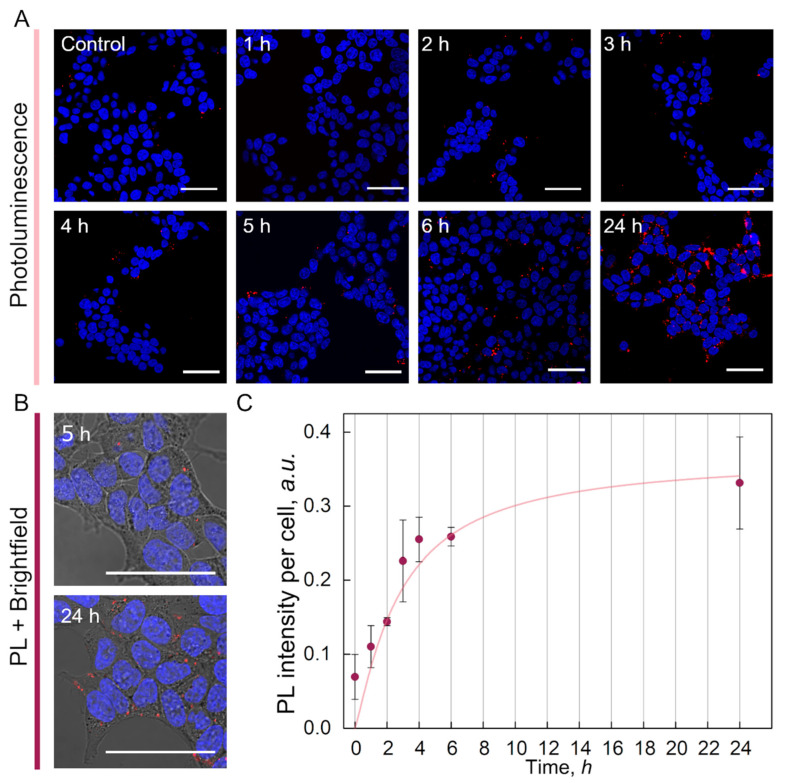
(**A**)—Confocal microscopy images of HEK-293T cells after different periods of incubation with ^99m^Tc-BSA-Au NCs. (**B**)—Confocal microscopy images of ^99m^Tc-BSA-Au NCs localization in HEK-239T cells. (**C**)—Photoluminescence intensity per cell of HEK-293T cells incubated with ^99m^Tc-BSA-Au NCs. At 0 h autofluorescence of control cells is displayed. Intensity values were calculated as mean ± standard deviation (N = 3, n = 3). Line is a guide to the eye.

A cellular accumulation study in human embryonic kidney HEK-293T cells was performed to determine whether ^99m^Tc-BSA-Au NCs could penetrate cells plasma membranes (Figure 6). We observed that up to 3 h incubation the accumulation is very low—only individual cells internalized ^99m^Tc-BSA-Au NCs (Figure 6A). After 4 to 6 h higher amounts of ^99m^Tc-BSA-Au NCs were already visible within the cells area. Finally, ^99m^Tc-BSA-Au NCs were efficiently accumulated inside HEK-293T cancer cells after 24 h of incubation (Figure 6A). No photoluminescence of ^99m^Tc-BSA-Au NCs was observed in the nuclei of the cells, nanoclusters were localized in the cytoplasm of cells (Figure 6B). Furthermore, to quantify the ^99m^Tc-BSA-Au NCs accumulation in cells, spectroscopic evaluation by measuring emission intensity of cell suspensions was performed. The results confirmed that uptake of Au NCs within HEK-293T cells increased with each hour of incubation. According to the accumulation dynamics curve, after 6 h of incubation the saturation process of ^99m^Tc-BSA-Au NCs PL signal within the cells began and after 24 h it reached saturation (Figure 6C). These findings are consistent with previous research of BSA-Au NCs on other cell lines, which found that saturation is reached after 6 h of and that longer incubation times do not significantly increase photoluminescence intensity per cell [27]. 

Cellular accumulation study in HEK-293T cells demonstrated that internalization of ^99m^Tc-BSA-Au NCs into cells is a slow process and takes several hours (Figure 6), whereas clearance of ^99m^Tc-BSA-Au NCs from bloodstream is fast and begins minutes after intravenous injection (Figure 3). Confocal imaging of histological sections revealed that there was no PL signal of ^99m^Tc-BSA-Au NCs in tissues even after 24 h after injection (Figure 5). Thus, our findings demonstrate that ^99m^Tc-BSA-Au NCs penetration from the bloodstream to tissues is limited.

**Figure 7:**
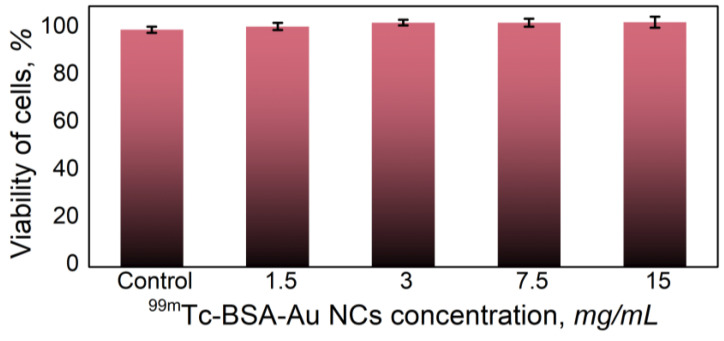
Viability of HEK-293T cells after 24 h of incubation with different concentrations of ^99m^Tc-BSA-Au NCs. There were no statistically significant changes compared to control.

Previous studies have shown that BSA-Au NCs are non-toxic to the cells [27,54,55,56]. In order to evaluate if the addition of ^99m^Tc could have any effects for cell viability, we assessed the cytotoxicity of ^99m^Tc-BSA-Au NCs to kidney cells. HEK-293T cells were treated with 1.5, 3, 7.5 or 15 mg/mL of ^99m^Tc-BSA-Au NCs for 24 h. LDH detection assay indicated that ^99m^Tc-BSA-Au NCs are non-toxic for the HEK-293T cells as their viability remained close to 100% even after 24 h of treatment with the concentration of 15 mg/mL of ^99m^Tc-BSA-Au NCs (Figure 7). 

## 4. Conclusions

Overall, our study evaluated ^99m^Tc-BSA-Au NCs as SPECT/PL dual-imaging probe distribution in vivo. Briefly, results demonstrated that attachment of ^99m^Tc to the BSA-Au NCs did not affect the optical properties of the ^99m^Tc-BSA-Au NCs. Over the first 2 h post-labelling ^99m^Tc-BSA-Au NCs yielded radiochemical purities of more than 95% (comparable to that obtained originally), indicating that in vivo imaging can be performed for at least 2 h. ^99m^Tc-BSA-Au NCs are stable in plasma for at least 3 days, exhibiting their eligibility for in vivo application. In vivo imaging of the Wistar rat, demonstrated intense cardiac blood pool activity, with fast blood clearance and rapid accumulation of ^99m^Tc-BSA-Au NCs in the kidneys, liver and urinary bladder, consistent with primarily renal excretion. However, there was no accumulation of ^99m^Tc-BSA-Au NCs in kidney, liver and spleen tissues after 2 and 24 h post injection. Cellular studies demonstrated that ^99m^Tc-BSA-Au NCs accumulation in kidney cells is a slow process, thus probably ^99m^Tc-BSA-Au NCs are cleared from the bloodstream faster than it can accumulate in tissues. Therefore, ^99m^Tc-BSA-Au NCs could be used as a SPECT/PL agent for bloodstream imaging of excretory organs, since they are stable enough for diagnostic purpose, are non-toxic and are easily eliminated from organism via urinary excretion system. 

## Supplementary Materials

The following supporting information can be downloaded at: https://www.mdpi.com/article/10.3390/nano12193259/s1, Figure S1: ^99m^Tc-Au-BSA NCs binding stability over time. Stability of ^99m^Tc-Au-BSA NCs stock solution and ^99m^Tc-Au-BSA NCs diluted in Wistar rat’s blood plasma is compared. Figure S2: Photoluminescence spectra of ^99m^Tc-BSA-Au NCs and normalized photoluminescence spectra of ^99m^Tc-BSA-Au NCs dispersed in rat’s blood plasma measured every 3 h. Changes of maximum PL spectral band intensity and maximum position (λ_max_) during time. References [12,29,40] are cited in the Supplementary Materials.

## References

[1] Weissleder R., Mahmood U. (2001). Molecular Imaging. Radiology.

[2] Chen K., Chen X. (2010). Design and Development of Molecular Imaging Probes. Curr. Top. Med. Chem..

[3] Massoud T.F., Gambhir S.S. (2003). Molecular Imaging in Living Subjects: Seeing Fundamental Biological Processes in a New Light. Genes Dev..

[4] James M.L., Gambhir S.S. (2012). A Molecular Imaging Primer: Modalities, Imaging Agents, and Applications. Physiol. Rev..

[5] Xing Y., Zhao J., Conti P.S., Chen K. (2014). Radiolabeled Nanoparticles for Multimodality Tumor Imaging. Theranostics.

[6] Mezzanotte L., van ’t Root M., Karatas H., Goun E.A., Löwik C.W.G.M. (2017). In Vivo Molecular Bioluminescence Imaging: New Tools and Applications. Trends Biotechnol..

[7] Wu M., Shu J. (2018). Multimodal Molecular Imaging: Current Status and Future Directions. Contrast Media Mol. Imaging.

[8] Walter A., Paul-Gilloteaux P., Plochberger B., Sefc L., Verkade P., Mannheim J.G., Slezak P., Unterhuber A., Marchetti-Deschmann M., Ogris M. (2020). Correlated Multimodal Imaging in Life Sciences: Expanding the Biomedical Horizon. Front. Phys..

[9] Azhdarinia A., Ghosh P., Ghosh S., Wilganowski N., Sevick-Muraca E.M. (2012). Dual-Labeling Strategies for Nuclear and Fluorescence Molecular Imaging: A Review and Analysis. Mol. Imaging Biol..

[10] Xie J., Zheng Y., Ying J.Y. (2009). Protein-Directed Synthesis of Highly Fluorescent Gold Nanoclusters. J. Am. Chem Soc..

[11] Kubiliute R., Slektaite A., Burkanas M., Grigiene R., Rotomskis R., Adliene D. (2011). Gold Nanoparticles as a Contrast Agent for X-ray Imaging. Proceedings of the Medical Physics in the Baltic States.

[12] Poderys V., Matulionytė-Safinė M., Rupšys D., Rotomskis R. (2016). Protein Stabilized Au Nanoclusters: Spectral Properties and Photostability. Lith. J. Phys..

[13] Su D., Gao L., Gao F., Zhang X., Gao X. (2020). Peptide and Protein Modified Metal Clusters for Cancer Diagnostics. Chem. Sci..

[14] Cifuentes-Rius A., Deepagan V.G., Xie J., Voelcker N.H. (2021). Bright Future of Gold Nanoclusters in Theranostics. ACS Appl. Mater. Interfaces.

[15] Combes G.F., Vučković A.-M., Perić Bakulić M., Antoine R., Bonačić-Koutecky V., Trajković K. (2021). Nanotechnology in Tumor Biomarker Detection: The Potential of Liganded Nanoclusters as Nonlinear Optical Contrast Agents for Molecular Diagnostics of Cancer. Cancers.

[16] Gao Q., Zhang J., Gao J., Zhang Z., Zhu H., Wang D. (2021). Gold Nanoparticles in Cancer Theranostics. Front. Bioeng. Biotechnol..

[17] Cole L.E., Ross R.D., Tilley J.M., Vargo-Gogola T., Roeder R.K. (2015). Gold Nanoparticles as Contrast Agents in X-Ray Imaging and Computed Tomography. Nanomedicine.

[18] Zhang A., Tu Y., Qin S., Li Y., Zhou J., Chen N., Lu Q., Zhang B. (2012). Gold Nanoclusters as Contrast Agents for Fluorescent and X-Ray Dual-Modality Imaging. J. Colloid Interface Sci..

[19] Fang X., Wang Y., Ma X., Li Y., Zhang Z., Xiao Z., Liu L., Gao X., Liu J. (2017). Mitochondria-Targeting Au Nanoclusters Enhance Radiosensitivity of Cancer Cells. J. Mater. Chem. B.

[20] Jarockyte G., Poderys V., Barzda V., Karabanovas V., Rotomskis R. (2022). Blood Plasma Stabilized Gold Nanoclusters for Personalized Tumor Theranostics. Cancers.

[21] Poderys V., Jarockyte G., Bagdonas S., Karabanovas V., Rotomskis R. (2020). Protein-Stabilized Gold Nanoclusters for PDT: ROS and Singlet Oxygen Generation. J. Photochem. Photobiol. B Biol..

[22] Li L., Liu X., Fu C., Tan L., Liu H. (2015). Biosynthesis of Fluorescent Gold Nanoclusters for in Vitro and in Vivo Tumor Imaging. Opt. Commun..

[23] Ahmed S., Annu, Ikram S., Yudha S.S. (2016). Biosynthesis of Gold Nanoparticles: A Green Approach. J. Photochem. Photobiol. B Biol..

[24] El-Sayed N., Schneider M. (2020). Advances in Biomedical and Pharmaceutical Applications of Protein-Stabilized Gold Nanoclusters. J. Mater. Chem. B.

[25] Chen J., Chen Q., Liang C., Yang Z., Zhang L., Yi X., Dong Z., Chao Y., Chen Y., Liu Z. (2017). Albumin-Templated Biomineralizing Growth of Composite Nanoparticles as Smart Nano-Theranostics for Enhanced Radiotherapy of Tumors. Nanoscale.

[26] Yoo D., Lee D. (2017). Oligochitosan-Stabilized Photoluminescent Gold Nanoconstructs for Optical Bioimaging. Biomater. Res..

[27] Matulionyte M., Dapkute D., Budenaite L., Jarockyte G., Rotomskis R. (2017). Photoluminescent Gold Nanoclusters in Cancer Cells: Cellular Uptake, Toxicity, and Generation of Reactive Oxygen Species. Int. J. Mol. Sci.

[28] Xu C., Wang Y., Zhang C., Jia Y., Luo Y., Gao X. (2017). AuGd Integrated Nanoprobes for Optical/MRI/CT Triple-Modal in Vivo Tumor Imaging. Nanoscale.

[29] Wang Y., Xu C., Zhai J., Gao F., Liu R., Gao L., Zhao Y., Chai Z., Gao X. (2015). Label-Free Au Cluster Used for in Vivo 2D and 3D Computed Tomography of Murine Kidneys. Anal. Chem..

[30] Shen D., Henry M., Trouillet V., Comby-Zerbino C., Bertorelle F., Sancey L., Antoine R., Coll J.-L., Josserand V., Le Guével X. (2017). Zwitterion Functionalized Gold Nanoclusters for Multimodal near Infrared Fluorescence and Photoacoustic Imaging. APL Mater..

[31] Liang G., Xiao L. (2017). Gd3+-Functionalized Gold Nanoclusters for Fluorescence–Magnetic Resonance Bimodal Imaging. Biomater. Sci..

[32] Han L., Xia J.-M., Hai X., Shu Y., Chen X.-W., Wang J.-H. (2017). Protein-Stabilized Gadolinium Oxide-Gold Nanoclusters Hybrid for Multimodal Imaging and Drug Delivery. ACS Appl. Mater. Interfaces.

[33] Han W., Yang W., Gao F., Cai P., Wang J., Wang S., Xue J., Gao X., Liu Y. (2020). Iodine-124 Labeled Gold Nanoclusters for Positron Emission Tomography Imaging in Lung Cancer Model. J. Nanosci. Nanotechnol..

[34] Ljungberg M., Pretorius P.H. (2018). SPECT/CT: An Update on Technological Developments and Clinical Applications. Br. J. Radiol.

[35] Mariani G., Bruselli L., Kuwert T., Kim E.E., Flotats A., Israel O., Dondi M., Watanabe N. (2010). A Review on the Clinical Uses of SPECT/CT. Eur. J. Nucl. Med. Mol. Imaging.

[36] Hoogendam J.P., Veldhuis W.B., Hobbelink M.G.G., Verheijen R.H.M., van den Bosch M.A.A.J., Zweemer R.P. (2015). ^99m^Tc SPECT/CT Versus Planar Lymphoscintigraphy for Preoperative Sentinel Lymph Node Detection in Cervical Cancer: A Systematic Review and Metaanalysis. J. Nucl. Med..

[37] Togami S., Kawamura T., Yanazume S., Kamio M., Kobayashi H. (2020). Comparison of Lymphoscintigraphy and Single Photon Emission Computed Tomography with Computed Tomography (SPECT/CT) for Sentinel Lymph Node Detection in Endometrial Cancer. Int. J. Gynecol. Cancer.

[38] Wang Y.-F., Chuang M.-H., Chiu J.-S., Cham T.-M., Chung M.-I. (2007). On-Site Preparation of Technetium-99m Labeled Human Serum Albumin for Clinical Application. Tohoku J. Exp. Med..

[39] Wang Y.-F., Chen Y.-C., Li D.-K., Chuang M.-H. (2011). Technetium-99m-Labeled Autologous Serum Albumin: A Personal-Exclusive Source of Serum Component. J. Biomed. Biotechnol..

[40] Schneider C.A., Rasband W.S., Eliceiri K.W. (2012). NIH Image to ImageJ: 25 Years of Image Analysis. Nat. Methods.

[41] Ali M., Kumar A., Kumar M., Pandey B.N. (2016). The Interaction of Human Serum Albumin with Selected Lanthanide and Actinide Ions: Binding Affinities, Protein Unfolding and Conformational Changes. Biochimie.

[42] Marenco M., Canziani L., De Matteis G., Cavenaghi G., Aprile C., Lodola L. (2021). Chemical and Physical Characterisation of Human Serum Albumin Nanocolloids: Kinetics, Strength and Specificity of Bonds with ^99m^Tc and 68Ga. Nanomaterials.

[43] Rodewald R., Karnovsky M.J. (1974). Porous Substructure of the Glomerular Slit Diaphragm in the Rat and Mouse. J. Cell Biol..

[44] Wartiovaara J., Ofverstedt L.-G., Khoshnoodi J., Zhang J., Mäkelä E., Sandin S., Ruotsalainen V., Cheng R.H., Jalanko H., Skoglund U. (2004). Nephrin Strands Contribute to a Porous Slit Diaphragm Scaffold as Revealed by Electron Tomography. J. Clin. Investig..

[45] Tojo A., Onozato M.L., Kitiyakara C., Kinugasa S., Fukuda S., Sakai T., Fujita T. (2008). Glomerular Albumin Filtration through Podocyte Cell Body in Puromycin Aminonucleoside Nephrotic Rat. Med. Mol. Morphol..

[46] Hilliard L.M., Osicka T.M., Clavant S.P., Robinson P.J., Nikolic-Paterson D.J., Comper W.D. (2006). Characterization of the Urinary Albumin Degradation Pathway in the Isolated Perfused Rat Kidney. J. Lab. Clin. Med..

[47] Park C.H. (1988). Time Course and Vectorial Nature of Albumin Metabolism in Isolated Perfused Rabbit PCT. Am. J. Physiol..

[48] Zhang X.-D., Wu D., Shen X., Liu P.-X., Fan F.-Y., Fan S.-J. (2012). In Vivo Renal Clearance, Biodistribution, Toxicity of Gold Nanoclusters. Biomaterials.

[49] Chiu N.T., Lee B.F., Hwang S.J., Chang J.M., Liu G.C., Yu H.S. (2001). Protein-Losing Enteropathy: Diagnosis with (99m)Tc-Labeled Human Serum Albumin Scintigraphy. Radiology.

[50] Furtado A.K., Cabral V.L.R., Santos T.N., Mansour E., Nagasako C.K., Lorena S.L., Pereira-Filho R.A. (2012). Giardia Infection: Protein-Losing Enteropathy in an Adult with Immunodeficiency. World J. Gastroenterol..

[51] Brouwer O.R., Buckle T., Vermeeren L., Klop W.M.C., Balm A.J.M., van der Poel H.G., van Rhijn B.W., Horenblas S., Nieweg O.E., van Leeuwen F.W.B. (2012). Comparing the Hybrid Fluorescent-Radioactive Tracer Indocyanine Green-^99m^Tc-Nanocolloid with ^99m^Tc-Nanocolloid for Sentinel Node Identification: A Validation Study Using Lymphoscintigraphy and SPECT/CT. J. Nucl. Med..

[52] KleinJan G.H., van Werkhoven E., van den Berg N.S., Karakullukcu M.B., Zijlmans H.J.M.a.A., van der Hage J.A., van de Wiel B.A., Buckle T., Klop W.M.C., Horenblas S. (2018). The Best of Both Worlds: A Hybrid Approach for Optimal Pre- and Intraoperative Identification of Sentinel Lymph Nodes. Eur. J. Nucl. Med. Mol. Imaging.

[53] Zuckier L.S., Dohan O., Li Y., Chang C.J., Carrasco N., Dadachova E., Dohan O. (2004). Kinetics of Perrhenate Uptake and Comparative Biodistribution of Perrhenate, Pertechnetate, and Iodide by NaI Symporter-Expressing Tissues in Vivo. J. Nucl. Med..

[54] Escudero-Francos M.A., Cepas V., González-Menéndez P., Badía-Laíño R., Díaz-García M.E., Sainz R.M., Mayo J.C., Hevia D. (2017). Cellular Uptake and Tissue Biodistribution of Functionalized Gold Nanoparticles and Nanoclusters. J. Biomed. Nanotechnol..

[55] Zhang Z., Yao Y., Yuan Q., Lu C., Zhang X., Yuan J., Hou K., Zhang C., Du Z., Gao X. (2020). Gold Clusters Prevent Breast Cancer Bone Metastasis by Suppressing Tumor-Induced Osteoclastogenesis. Theranostics.

[56] Ungor D., Barbasz A., Czyżowska A., Csapó E., Oćwieja M. (2021). Cytotoxicity Studies of Protein-Stabilized Fluorescent Gold Nanoclusters on Human Lymphocytes. Colloids Surf. B Biointerfaces.

