# Supplementary material for: Biodistribution of Multimodal Gold Nanoclusters Designed for Photoluminescence-SPECT/CT Imaging and Diagnostic
Source: Nanomaterials (Basel). 2022 Sep 20;12(19):3259. doi: 10.3390/nano12193259 (PMC9565908; doi:10.3390/nano12193259)
Supplement: Supplementary file 1 [file nanomaterials-12-03259-s001.zip › nanomaterials-1924952-supplementary.pdf]

Supplementary Materials

Biodistribution of multimodal gold nanoclusters designed for luminescence-SPECT/CT imaging & diagnostic

Greta Jarockyte[1,2], Marius Stasys[1], Vilius Poderys[1], Kornelija Buivydaite[1,2], Marijus Pleckaitis[1,2], Danute Bulotiene[1], Marija Matulionyte[1], Vitalijus Karabanovas[1,3]*, Ricardas Rotomskis[1,4]

1 Biomedical Physics Laboratory, National Cancer Institute, LT-08406, Vilnius, Lithuania; greta.jarockyte@nvi.lt (G.J.); vilius.poderys@nvi.lt (V.P.); danute.bulotiene@nvi.lt (D.B.); marijus.pleckaitis@nvi.lt (M.P.); vitalijus.karabanovas@nvi.lt (V.K.); ricardas.rotomskis@nvi.lt (R.R.);
2 Life Sciences Center, Vilnius University, LT- 10257, Vilnius, Lithuania;
3 Department of Chemistry and Bioengineering, Vilnius Gediminas Technical University, LT-10223 Vilnius, Lithuania.
4 Laser Research Center, Faculty of Physics, Vilnius University, LT-10223 Vilnius, Lithuania.
* Correspondence: vitalijus.karabanovas@nvi.lt

Binding stability of ^{99m}Tc-Au-BSA NCs

Binding stability of ^{99m}Tc-Au-BSA NCs was evaluated measuring the thin-layer chromatography strips with a Siemens Symbia T6 gamma camera with a Low Energy High Resolution (LEHR) collimator, placing a strip on the detector, and acquiring a 5 min static image. Quantitative analysis of the images based on the counts in regions of interest (ROIs) was carried out using ImageJ software [1].

The stability of the radiolabeled nanoparticle was checked by repeating the measurements over 2 h period and after 12 h. It was estimated that within the first two hours, the radiochemical purity of the ^{99m}Tc-Au-BSA NCs remains above 97 % as the initial solution (Figure S1). It should be mentioned, that at 12 h post preparation the amount of radiolabeled nanoparticles has decreased by a quarter. There were no differences observed comparing binding stability of stock solution and ^{99m}Tc-Au-BSA NCs diluted in Wistar rat's blood plasma. Results presented in Figure S1 suggest that the time of the imaging procedure with ^{99m}Tc-Au-BSA NCs should not exceed 2 h.

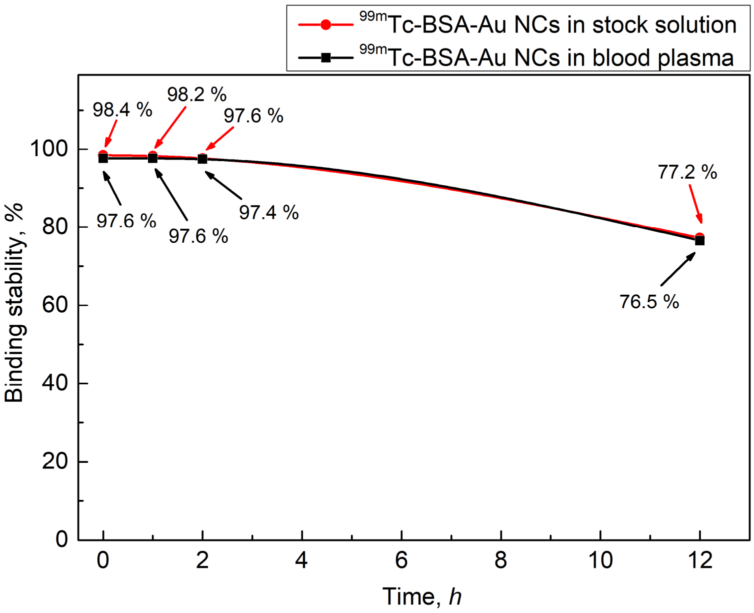

Figure S1. ^{99m}Tc-Au-BSA NCs binding stability over time. Stability of ^{99m}Tc-Au-BSA NCs stock solution and ^{99m}Tc-Au-BSA NCs diluted in Wistar rat's blood plasma is compared.

Colloidal stability in blood plasma

Our previous study demonstrated that BSA-Au NCs are colloidally stable for more than a month in stock solution after synthesis [2]. Also, it was showed, that BSA-Au NCs are stable solved in 10 % FBS buffer solution at least for 24 h [3]. However, there is not enough information about ^{99m}Tc-BSA-Au NCs stability in biological media, thus we investigated ^{99m}Tc-BSA-Au NCs stability in Wistar rat's blood plasma before study with experimental animals. In Figure S2 changes of photoluminescence spectra of ^{99m}Tc-BSA-Au NCs are demonstrated. PL intensity have increased over time (Figure S2 A, C), while shape of PL spectrum and λ_{max} remained unchanged (Figure S2 B, D). Thus, prepared ^{99m}Tc-BSA-Au NCs are stable in rat's blood plasma and are suitable for *in vivo* studies.

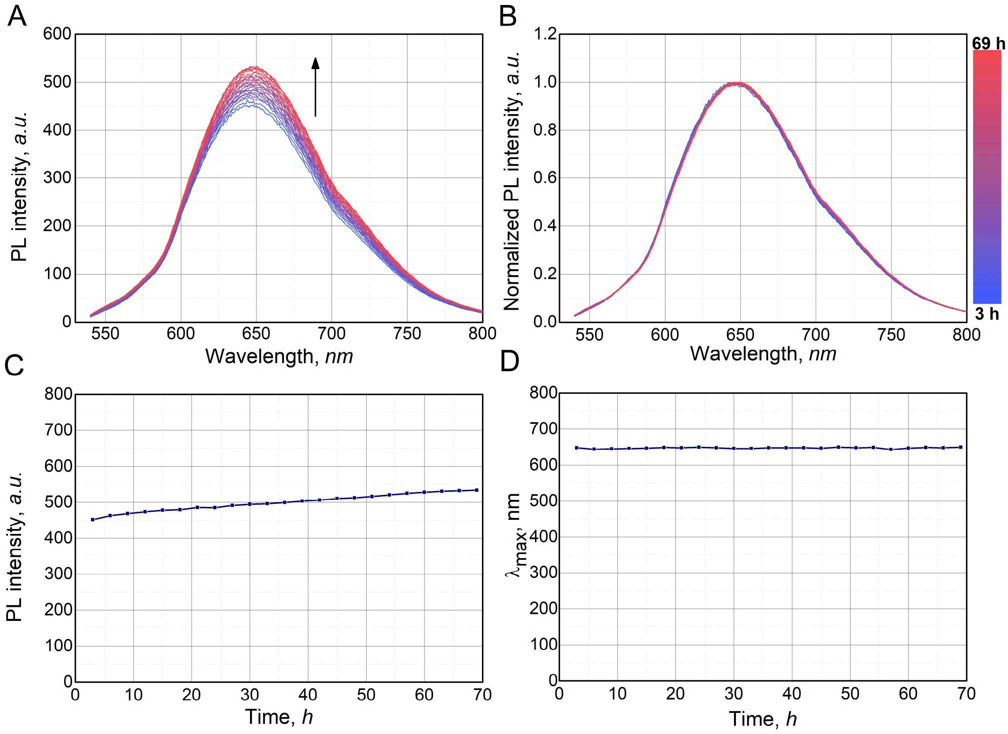

Figure S2. Photoluminescence spectra of ^{99m}Tc-BSA-Au NCs (A) and normalized photoluminescence spectra of ^{99m}Tc-BSA-Au NCs (B) dispersed in rat's blood plasma measured every 3 h. Changes of maximum PL spectral band intensity (C) and maximum position (λ_{max}) (D) during time.

References

1. Schneider, C.A.; Rasband, W.S.; Eliceiri, K.W. NIH Image to ImageJ: 25 Years of Image Analysis. *Nat Methods* **2012**, *9*, 671–675, doi:10.1038/nmeth.2089.
2. Poderys, V.; Matulionytė-Safinė, M.; Rupšys, D.; Rotomskis, R. Protein Stabilized Au Nanoclusters: Spectral Properties and Photostability. *Lithuanian Journal of Physics* **2016**, *56*, 55–65, doi:10.3952/physics.v56i1.3277.
3. Wang, Y.; Xu, C.; Zhai, J.; Gao, F.; Liu, R.; Gao, L.; Zhao, Y.; Chai, Z.; Gao, X. Label-Free Au Cluster Used for in Vivo 2D and 3D Computed Tomography of Murine Kidneys. *Anal. Chem.* **2015**, *87*, 343–345, doi:10.1021/ac503887c.
